# Reply to Kato et al. Comment on “Sokawa, Y. Radiation-Induced Childhood Thyroid Cancer after the Fukushima Daiichi Nuclear Power Plant Accident. *Int. J. Environ. Res. Public Health* 2024, *21*, 1162”

**DOI:** 10.3390/ijerph22050675

**Published:** 2025-04-25

**Authors:** Yoshihiro Sokawa

**Affiliations:** Department of Applied Biology, Kyoto Institute of Technology, Kyoto 606-8585, Japan; sokawa@snr.kit.ac.jp

I thank you for considering my paper [1]. In the paper, I presented the following four points [2]:

1. There were clear regional differences in the incidence of childhood thyroid cancer after the Fukushima Nuclear Power Plant accident. Radiation from the accident caused the development of cancer.
2. The rate of “Common Case” before and after the accident was obtained from the results of the Basic Survey (BS) and the first Full-Scale Survey (FSS).
3. The “Radiation-induced Case” of childhood thyroid cancer was found to have two waves: the first wave lasted up to about 6 years after the accident, and then the second wave followed.
4. The first wave might have been caused by the destruction of the immune system due to radiation exposure, and the second wave by genetic mutation due to exposure in early childhood.

Cancer statistics only show the results of incidental detection through voluntary medical examinations by individuals [3]. On the other hand, after the nuclear power plant accident, Fukushima Prefecture conducted a mass screening using echoes for all persons under 18 years of age [2]. As with school and workplace examinations, group examinations involve a certain degree of coercion. In contrast, the cancer registry counts thyroid cancers that were found by chance after visiting a hospital for some physical ailment. Since the purpose and detection methods of the cancer statistics and the Fukushima screening are completely different, it is not possible to directly compare the detection rates of the two.

Each of the four cumulative lines of areas A, B, C, and D shown in Figure 1 of my paper intersected at almost the same point on the *Y* axis. That is unlikely to be coincidental, and it shows that the data processing was appropriate. The data were based on the reports published by the Fukushima Health Management Survey [4,5,6,7,8].

So far, the BRAF^V600E^ mutation, which is found in adults, has been detected in BS and first FSS patients, and the chromosomal rearrangements RET/PTC3, which were observed in Chernobyl, have not been observed in Fukushima [9]. The Fukushima Medical University is expected to investigate the possibility of finding mutations other than the BRAF^V600E^ mutation, such as the chromosomal rearrangements RET/PTC3, in patients observed in the third and fourth FSS.
